# The Throat

**Published:** 1886-09

**Authors:** 


### *THE THROAT.*

The throat is a wonderful instrument of music. Place the fingers upon it, and every time you speak you can feel the vibration of the vocal organs, producing sound. Anything that even *touches* the throat impairs the purity of those sounds. Fling a cloth over the strings of a piano or a violin, and get music out of it if you can. So every cloth which surrounds the throat impairs the sweetness of the voice. Women go with necks bare; men have theirs swathed and bandaged, and ten women have sweet voices where one man has. A man's voice should be as pure as a woman's. Why is it not? He is shaved and choked. God has provided a covering for man's throat, light and soft; it clothes the neck and preserves the health. But a man gets a sharp iron, scrapes his neck, ties a rag around it, takes cold, has sore throat, bronchitis, consumption, and dies.—*Christian Advocate.*

